# Sensitivity and Specificity of Point-of-Care Rapid Combination Syphilis-HIV-HCV Tests

**DOI:** 10.1371/journal.pone.0112190

**Published:** 2014-11-06

**Authors:** Kristen L. Hess, Dennis G. Fisher, Grace L. Reynolds

**Affiliations:** Center for Behavioral Research and Services, California State University Long Beach, Long Beach, California, United States of America; Johns Hopkins Bloomberg School of Public Health, United States of America

## Abstract

**Background:**

New rapid point-of-care (POC) tests are being developed that would offer the opportunity to increase screening and treatment of several infections, including syphilis. This study evaluated three of these new rapid POC tests at a site in Southern California.

**Methods:**

Participants were recruited from a testing center in Long Beach, California. A whole blood specimen was used to evaluate the performance of the Dual Path Platform (DPP) Syphilis Screen & Confirm, DPP HIV-Syphilis, and DPP HIV-HCV-Syphilis rapid tests. The gold-standard comparisons were *Treponema pallidum* passive particle agglutination (TPPA), rapid plasma reagin (RPR), HCV enzyme immunoassay (EIA), and HIV-1/2 EIA.

**Results:**

A total of 948 whole blood specimens were analyzed in this study. The sensitivity of the HIV tests ranged from 95.7–100% and the specificity was 99.7–100%. The sensitivity and specificity of the HCV test were 91.8% and 99.3%, respectively. The treponemal-test sensitivity when compared to TPPA ranged from 44.0–52.7% and specificity was 98.7–99.6%. The non-treponemal test sensitivity and specificity when compared to RPR was 47.8% and 98.9%, respectively. The sensitivity of the Screen & Confirm test improved to 90.0% when cases who were both treponemal and nontreponemal positive were compared to TPPA+/RPR ≥1∶8.

**Conclusions:**

The HIV and HCV on the multi-infection tests showed good performance, but the treponemal and nontreponemal tests had low sensitivity. These results could be due to a low prevalence of active syphilis in the sample population because the sensitivity improved when the gold standard was limited to those more likely to be active cases. Further evaluation of the new syphilis POC tests is required before implementation into testing programs.

## Introduction

Worldwide, syphilis remains a large problem with an estimated 11 million new cases in adults in 2005 [1]. The majority of these cases occur in developing countries. Since 2000 there has been an increase in the number of syphilis cases in the United States, predominately among men who have sex with men (MSM) [2]. The standard method for syphilis diagnosis requires two screening tests, a non-treponemal test such as the rapid plasma reagin (RPR) or the Venereal Diseases Research Laboratory (VDRL) test, and a treponemal test such as *T. pallidum* passive particle agglutination (TPPA) or fluorescent treponemal antibody absorption (FTA-ABS) [3]. Traditionally, a treponemal test is only run after a positive non-treponemal test is obtained. Recently, an alternative algorithm has been adopted by some labs and hospitals where a treponemal test is completed first in the sequence [4]. However, the equipment and personnel needed to conduct the standard tests for syphilis can be prohibitively expensive in low-resource settings. In addition, the delay in receiving the test results can lead to missed treatment opportunities because of a failure to return for test results [5]–[7].

One solution to these issues with traditional testing is point-of-care (POC) rapid tests [8]. In recent years treponemal POC rapid tests have been developed and have been shown to have good sensitivity and specificity [9], [10]. However, these tests only detect the presence of treponemal antibodies, so they cannot distinguish between active cases and historical, treated cases. Recently, a dual rapid test has been developed that tests for treponemal antibodies and reagin (DPP Syphilis Screen and Confirm). A study on archived samples and a prospective study in China have both shown promising results [11], [12]. In addition, syphilis POC rapid tests have also been paired with tests for other infections. These tests detect the treponemal antibody as well as HIV infection in one test [13] and HIV and hepatitis C virus (HCV) in another. Given the high rate of co-infection and similar populations at risk [14], it would be advantageous to have a test that could simultaneously detect the presence of multiple infections. This study evaluated the performance of three rapid POC tests in an at-risk population seeking HIV and sexually transmitted infection (STI) testing at a testing center in Long Beach, CA.

## Methods

### Ethical Approval

The protocol was approved by the California State University Long Beach (CSULB), Institutional Review Board (IRB). All participants provided written informed consent using a form approved by the CSULB IRB that included permission to re-contact the participant to notify them of future studies they may be interested in.

### Participants

Data for this study were collected from May 26, 2011 to June 30, 2013. Participants were recruited from clients at the Center for Behavioral Research and Services (CBRS) in Long Beach, California. CBRS provides free HIV and STD testing to the community as well as conducts research. The pre-study HIV prevalence was 2.6%, the pre-study prevalence of HCV was 48.1%, the pre-study prevalence of RPR was 3.0%, and the pre-study prevalence of TP-PA was 8.1%. Clients who came in for HIV and other STI testing were screened for eligibility. In addition, existing clients who were eligible and whose last visit was more than three months prior were sent letters inviting them to come in for the study. Eligible clients were 15 years of age and older, had not participated previously, and reported being in a behavioral risk group. Behavioral risk groups were defined as: (1) injection drug users (IDU) with verified track marks (e.g., visible signs of injection) [15], (2) women who reported at least two male partners in the last two years or engaging in anal intercourse, sex trading, or sex with a man who has sex with men (MSM), an IDU, or an HIV positive man, (3) MSM and men who have sex with men and women (MSMW), and (4) transgender individuals. These definitions of the risk groups were based on guidelines from the Los Angeles County Department of Public Health. Clients were not excluded based on prior infection history. When an eligible client agreed to participate, they gave written informed consent, and a California state licensed phlebotomist drew a venous blood sample, by standard laboratory practices, for the POC tests, as well as the gold standard confirmatory tests. Every test was performed by the phlebotomist and was completed on the whole blood specimen of each participant. However, there was variation in sample size by test because not all experimental test kits were available at all times. During the study session, the participant also completed several questionnaires to gather demographic data as well as their impressions of the testing procedure, personality measures, and risk-behavior information. Two weeks after the initial visit, the participant returned for the gold-standard results. Participants were given the results of the POC tests, but it was stressed that these were experimental and that they should return in two weeks to obtain the gold-standard results. Participants were referred to care upon receiving a positive result from the gold standard test. Clinical diagnosis could not be obtained, therefore it was not possible to correlate it with the results of the laboratory testing.

This paper contains the results for the POC tests that included detection of syphilis infection. These were the Dual Path Platform (DPP) Syphilis Screen & Confirm Assay, the DPP HIV-Syphilis Assay, and the DPP HIV-HCV-Syphilis Assay. All three were manufactured by Chembio Diagnostic Systems, Inc. (Medford, NY). The test kits were stored in a temperature-controlled setting, with the temperature being both monitored and recorded. The test description has been described [12], [13]. The test procedures were based on the manufacturer’s venipuncture whole blood specimen instructions and the phlebotomists were trained in person on-site in Long Beach by Chembio Staff. For the Syphilis Screen & Confirm Assay, 10 µl of blood was added directly to well 1 followed by two drops of Running Buffer. After five minutes, five drops of Running Buffer were added to Well 2. The results were read 10 to 15 minutes after the addition of buffer to Well 2, but not more than 20 minutes from the addition of buffer to Well 1. There were digital timers in the phlebotomy laboratory that were set to the exact time requirements for the tests. If no control line appeared, the test was discarded. If a control line appeared, then the assay was assessed for the appearance of lines which indicated reactivity to treponemal and non-treponemal antibodies. The procedure for the HIV-Syphilis and HIV-HCV-Syphilis assays were similar to the Syphilis Screen & Confirm Assay, except that, for these two tests, the 10 µl of venous blood was added to the sample buffer bottle. The bottle was then gently shaken and two drops were added to Well 1. After five minutes, Running Buffer (with no blood) was added to Well 2. The results were read using the same method as the Syphilis Screen & Confirm Assay.

The sensitivity and specificity of the three test kits were determined by comparing the results to the gold-standard test results. The comparison test for the treponemal antibody test was TPPA (Fujirebio Inc., Tokyo, Japan) and the comparison for the non-treponemal test was RPR (ASI Arlington Scientific, Inc., Springville, UT). The gold standards for HCV and HIV were HCV enzyme immunoassay (EIA) 2.0 (Abbott Laboratories, Abbott Park, IL) and GS HIV-1/HIV-2 *PLUS O* EIA (Bio-Rad Laboratories, Redmond, WA), respectively. Analyses were performed with the SAS software package version 9.3 (Cary, NC). Sensitivity and specificity were obtained using PROC FREQ and exact confidence intervals were reported. All p-values reported were based on chi-square tests.

## Results

Over the course of the study, 2083 people were screened for eligibility. Of these, 859 were deemed not eligible. The majority of people were not eligible because they were not at increased risk for HIV, such as men with only female partners who do not inject drugs and women with only one male partner. Of those who were eligible, 142 were not offered enrollment in the study, which was typically due to time constraints. Of the people who were offered enrollment, 31 declined. The reasons for declining to participate included lack of time to complete the study visit and not wanting to receive test results the same day. The final sample size for this analysis was 948 after removing the 103 participants who were not able to give a blood sample or were missing RPR and TPPA test results. The sample was 54% men, 44% women, and 1.4% transgender. A third of the sample (35%) was non-Hispanic Black, 26% were Hispanic, and 26% were non-Hispanic White (Table 1). The average age was 39.9 years (*SD* = 12.7) with a range of 15–77 years. About one-fifth (22%) reported injecting drugs, 39% were women at sexual risk, and 37% were MSM/MSMW. The prevalence of HIV in the sample was 7% (*n* = 66), 18% (*n* = 166) tested positive for HCV, and 2% (*n* = 23) tested positive for TPPA and RPR. An additional 87 people tested positive for only TPPA and 4 tested positive only for RPR.

**Table 1:** Sample Demographics (*N* = 948).

Characteristic	*N*	%
Gender		
Male	516	54.4
Female	419	44.2
Transgender (Male to Female)	11	1.2
Transgender (Female to Male)	2	0.2
Race/Ethnicity		
Hispanic	250	26.4
White	243	25.6
Black	333	35.1
Asian	20	2.1
Hawaiian/Pacific Islander	7	0.7
Native American	11	1.2
More than 1 race reported	76	8.0
Behavioral Risk Group		
Injection drug user	210	22.2
Women at sexual risk	365	38.5
MSM/MSMW	355	37.4
Transgender	13	1.4
Results from Gold Standard tests		
HIV positive	66	7.0
Hepatitis C Virus positive	166	17.6
TPPA+RPR positive	23	2.4
TPPA+RPR ≥1∶8	10	1.1
Only TPPA positive	87	9.2
Only RPR positive	4	0.4

### DPP Syphilis Screen & Confirm Assay

The sensitivity of the non-treponemal test when compared to RPR positivity (titer 1∶1 or higher) was 47.8% (95% CI: 26.8–69.4%) and the specificity was 98.9% (95% CI: 97.9–99.5%; Table 2). The sensitivity improves to 57.9% when the gold standard comparison is TPPA and RPR positivity (i.e. remove the biologic false positives) and improves further to 90.0% when the comparison is TPPA positive and an RPR titer of 1∶8 or greater (i.e. the cases more likely to be active syphilis). The sensitivity of the test among those with an RPR titer of 1∶4 or less is significantly less than the sensitivity among those with a titer of 1∶8 or greater (22.2% vs. 90.0%, *p* = .003). The sensitivity is low for the treponemal test when compared to TPPA positivity, 52.7% (95% CI: 42.1–63.1%). The specificity of this test was 98.7% (95% CI: 97.5–99.4%; Table 2). When participants who were nontreponemal/treponemal rapid test positive were compared to TPPA+RPR ≥1∶8, the sensitivity was 90.0% (95% CI: 55.5–99.8%) and the specificity was 99.6% (95% CI: 98.8–99.9%).

**Table 2:** Sensitivity and Specificity of DPP Syphilis Screen and Confirm Assay.

	Gold Standard Results					
POC Test	RPR		TPPA+RPR		TPPA+RPR ≥1∶8	
**Non-Treponemal**	+	−	+	−	+	−
+	11	8	11	8	9	10
−	12	732	8	737	1	744
Sensitivity (%) (95% CI)	47.8 (26.8–69.4)		57.9 (33.5–79.8)		90.0 (55.5–99.8)	
Specificity (%) (95% CI)	98.9 (97.9–99.5)		98.9 (97.9–99.5)		98.7 (97.6–99.4)	
	**TPPA**		**TPPA+RPR**		**TPPA+RPR ≥1**∶**8**	
**Treponemal**	+	−	+	−	+	−
+	49	9	15	43	9	49
−	44	663	4	705	1	708
Sensitivity (%) (95% CI)	52.7 (42.1–63.1)		79.0 (54.4–94.0)		90.0 (55.5–99.8)	
Specificity (%) (95% CI)	98.7 (97.5–99.4)		94.3 (92.3–95.8)		93.5 (91.5–95.2)	
					**TPPA+RPR ≥1**∶**8**	
**Non-Treponemal+Treponemal**					+	−
+	–	–	–	–	9	3
−	–	–	–	–	1	753
Sensitivity (%) (95% CI)	–		–		90.0 (55.5–99.8)	
Specificity (%) (95% CI)	–		–		99.6 (98.8–99.9)	

*Note*: CI = Confidence interval.

### DPP HIV-HCV-Syphilis Assay

The sensitivities of the HIV-HCV-Syphilis tests were 100% (95% CI: 93.8–100%), 91.8% (95% CI: 86.3–95.6%), and 44.0% (95% CI: 34.8–54.3%; Table 3), respectively. The specificity of the HIV test was 99.9% (95% CI: 99.3–100%). The specificity of the HCV test was 99.3% (95% CI: 98.4–99.8%), and the specificity of the syphilis test was 99.4% (95% CI: 98.5–99.8%).

**Table 3:** Sensitivity and Specificity of DPP HIV-HCV-Syphilis Assay & DPP HIV-Syphilis Assay.

POC Test	Gold Standard		Sensitivity (%) (95% CI)	Specificity (%) (95% CI)
DPP HIV-HCV-Syphilis	+	−		
HIV				
+	58	1	100 (93.8–100)	99.9 (99.3–100)
−	0	813		
HCV				
+	145	5	91.8 (86.3–95.6)	99.3 (98.4–99.8)
−	13	703		
Syphilis (Treponemal)				
	44	5	44.0 (34.8–54.3)	99.4 (98.5–99.8)
−	56	776		
DPP HIV-Syphilis Assay (original order: HIV-syphilis)				
HIV				
+	18	0	100 (81.5–100)	100 (98.5–100)
−	0	244		
Syphilis (Treponemal)				
+	13	1	46.4 (27.5–66.1)	99.6 (97.7–100)
−	15	234		
DPP HIV-Syphilis Assay (reverse order: syphilis-HIV)				
HIV				
+	44	2	95.7 (85.2–99.5)	99.7 (98.8–100)
−	2	606		
Syphilis (Treponemal)				
+	37	3	47.4 (36.0–59.1)	99.5 (98.5–99.9)
−	41	576		

*Note*: CI = Confidence interval, Gold Standard for syphilis was TPPA.

### DPP HIV-Syphilis Assay

The original order of the tests in this assay was HIV first, syphilis second. The company switched the order in hopes of increasing the sensitivity of the syphilis test. Therefore, two different sets of sensitivities and specificities are presented. In the original test, the sensitivity and specificity of the syphilis test was 46.4% (95% CI: 27.5–66.1%) and 99.6% (95% CI: 97.7–100%; Table 3), respectively. After the test order was reversed, the sensitivity and specificity did not change, 47.4% (95% CI: 36.0–59.1%) and 99.5% (95% CI: 98.5–99.9%), respectively. The sensitivity and specificity of the HIV test was originally 100% (95% CI: 81.5–100%) and 100% (95% CI: 98.5–100%), respectively. After the change in configuration, the sensitivity was 95.7% (95% CI: 85.2–99.5%) and the specificity was 99.7% (95% CI: 98.8–100%).

We examined concordance of the treponemal result between the three POC tests. Among participants who had data for all three tests and had a positive result on at least one of the three (n = 55), 40 had a positive result on all three tests (73%). The concordance between the two POC tests that included HIV was 100%.

## Discussion

The multi-infection test kits had good sensitivity and specificity for HIV and HCV although the treponemal test had a poor sensitivity. A single rapid test that can simultaneously detect multiple infections would be very useful in the field, given the overlapping risk groups of these infections [14], [16]. The good sensitivity of the HIV and HCV on the HIV-HCV-Syphilis rapid test suggests that combining the tests onto one device is possible. However, the treponemal test in this case does not appear to be performing well. In addition, these multi-infection tests still have the drawback of only detecting the treponemal antibody making it difficult to distinguish between an active case and a treated case of syphilis. When combining the tests onto one device it may be advantageous to include both the treponemal and nontreponemal tests to avoid over diagnosis and treatment.

We found two other studies of the ChemBio Screen and Confirm syphilis test, the dual treponemal and non-treponemal test, a study by Yin et al. from China [11] and a study by Causer et al. from Australia [17]. Both of these studies found a better sensitivity than the current study. Our non-treponemal sensitivity value of 47.8 was significantly lower than the sensitivity in the Yin et al. study of 87.6, and our treponemal sensitivity of 52.7 was significantly lower than the Yin et al. value of 96.2 and the Causer et al. value of 89.8. One reason for the discrepancy in results could be due to error in test administration or result interpretation, but given the high sensitivities of the HIV and HCV rapid tests in the multi-infection test kits, and the fact that the phlebotomists were personally trained by company staff, the likelihood of this is low. A more plausible explanation is the low prevalence of high-titer syphilis in the sample population. In the current study the proportion of TPPA+ cases that were high titer was only 11%, which is significantly lower than the 50% prevalence in the other two samples. Indeed, when the gold-standard comparison included only cases with a high titer, the sensitivity of both the treponemal and nontreponemal lines improved greatly. The Yin et al. sample also showed a reduction in sensitivity of the non-treponemal test among lower titer cases. This suggests that the nontreponemal rapid test is not as sensitive to the lower titer samples. A little harder to explain is the low sensitivity of the treponemal results, because TPPA usually remains detectable for an individual’s lifetime after infection, but previous work has shown a decrease in TPPA concentration after treatment [18]. Potentially the TPPA concentration in treated cases has dipped below the detection level of the rapid POC test, but is still detectable on the gold-standard test.

The results of all three studies suggest that this POC test has adequate sensitivity with a case mix that has more high titers, and may not have adequate sensitivity with a case mix that has lower titers. Because the goal of the rapid POC test would be to detect active syphilis that requires treatment, the lower sensitivities on low titer and RPR negative samples may not be detrimental. However, we were not able to definitively determine which cases in our sample were treated and which were active, or the stage of infection among active cases because we were not able to obtain clinical diagnosis information. Therefore, some of the lower titer cases may have still been active cases requiring treatment.

This study included people at higher risk for HIV and STI infection, but there was still a low prevalence of syphilis infection. The sensitivity and specificity estimates would have been more precise if the sample had included more positive cases, but it is also important to determine the accuracy of the test in low prevalence settings if the rapid test is to be used across all types of populations. POC tests are an important new tool in the detection and treatment of infectious diseases [19], [20]. Despite the fact that we found low sensitivity for the treponemal and nontreponemal POC tests, the results are still promising because the POC test was able to detect a majority of the potentially active cases, which are the more important to diagnosis and treat. However, more studies should be conducted to better characterize these new POC tests.
